# Editorial: Metabolic regulation in immune processes against microbial infections

**DOI:** 10.3389/fimmu.2023.1320443

**Published:** 2023-10-27

**Authors:** Ming Jiang, Jie Yin, Cheng Zhixue, Hetron Mweemba Munang’andu

**Affiliations:** ^1^ State Key Laboratory of Swine and Poultry Breeding Industry, Guangdong Key Laboratory of Animal Breeding and Nutrition, Institute of Animal Science, Guangdong Academy of Agricultural Sciences, Guangzhou, China; ^2^ College of Animal Science and Technology, Hunan Agricultural University, Changsha, China; ^3^ Department of Pulmonary and Critical Care Medicine Third Affiliated Hospital of Sun Yat-sen University, Guangzhou, China; ^4^ Faculty of Biosciences and Aquaculture, Nord University, Bodø, Norway

**Keywords:** immune cells, defense, homeostasis, metabolic regulation, microbial infections

## Introduction

Immune cells undergo changes during infection, serving as defense cells, conducting microbial surveillance, and maintaining homeostasis. These processes are supported by metabolic regulations that provide the necessary energy and nutrients. Immune processes and metabolic regulation are highly integrated, but information on their interdependency is limited, which is a key discussion in this Research Topic. This editorial’s summary reveals a diverse collection of articles, covering topics such as nutritional regulation of immune responses, the gut microbiota-immune axis, virulence reduction using metabolites, development of attenuated live vaccines through the deletion of bacterial transcription factor genes, host reprogramming using metabolites as a protective measure against microbial infections, and the identification of endogenous compounds in host cells with potential as drug candidates against microbial infections.

## Nutrition and gut microbiota

Hu et al. reviewed the interaction between dietary fiber (DF) and gut microbiota in pigs. They underscored the pivotal role of DF in microbial fermentation, generating short-chain fatty acids that serve as the primary energy source. Additionally, DF plays a vital role in upholding normal intestinal function, homeostasis, and immunomodulation, thus mitigating infection and inflammation. Their assertions were substantiated by referencing pertinent studies (1, 2) that illustrate how manipulating DF can reshape the gut microbiota, ultimately promoting porcine health. Conversely, inadequate DF can perturb the microbiota equilibrium, potentially leading to pathogen-induced damage to the intestinal mucosa. Therefore, the manipulation of DF presents a means to mitigate symbiosis, attain specific microbial populations, and restore gut homeostasis. In a distinct investigation, Ding et al. compared the ability of indigenous Chinese pig breeds, Taoyuan black (TB) and Xiangcun black (XB), with that of the exotic Duroc (DR) breed in modulating intestinal immunity and barrier function. All three breeds were subjected to diets with varying DF levels, including low and high DF regimens. The study unveiled that DF regulation impacted the plasma immune cells of TB and DR pigs, while XB pigs exhibited enhanced barrier function. It was noted that DR pigs exhibited heightened ideal inflammation, suggesting that indigenous Chinese pig breeds, TB and XB, displayed greater tolerance to DF variations compared to the exotic DR pigs. Continuing on to another study, Zhou et al. explored the effects of restricting crude protein (CP) and metabolizable energy (ME) on the expression of immunoglobulins (Igs) in serum and immune genes in the spleen of broilers fed diets with low, standard, and high ME or CP content. Their findings revealed significantly lower IgM levels in the group on a low ME diet, in contrast to the other groups. Simultaneously, immune genes like *Mx1, UPS18, TLR4, IFNγ*, and *IL-18* were significantly upregulated in the group exposed to reduced dietary density. This study underscores how varying nutritional density can differentially impact the immune status of chickens.

## Vaccine development, virulence attenuation and antimicrobial resistance

Vaccination, a widely employed and effective strategy for disease control, has seen less investigation into the metabolic aspect of host response compared to the extensively studied immune responses, commonly used as indicators of vaccine protection (3, 4). This has left the question unanswered regarding whether metabolic responses can serve as a metric for vaccine protection and contribute to improving vaccine efficacy. However, Wang et al. demonstrated that co-administering exogenous fructose with a live *Edwasiella tarda* vaccine significantly increased the survival rates of vaccinated fish and mice following a challenge. In a separate study, Xiang et al. showed that exogenous aspartate provided protection to Zebrafish (*Danio rerio*) against infections caused by both tetracycline-sensitive and resistant *E. tarda*. Given that exogenous aspartate promotes nitric oxide (NO) biosynthesis, a known enhancer of innate immunity against bacterial infections, they replaced aspartate with sodium nitroprusside to induce NO production, achieving similar levels of protection against the same bacteria as observed with aspartate. Collectively, these studies highlight the potential use of exogenous metabolites in host reprogramming to provide protection against antimicrobial-resistant bacteria. In the context of vaccine development, these metabolites can be harnessed to enhance vaccine efficacy. Yet, Zhang et al. revealed that the deletion of the *orf02889* gene, encoding the *AraC* transcription factor in *Aeromonas hydrophila* strain LP-2, significantly reduced its virulence. This genetic modification serves as an attenuation method in the development of live vaccines. Furthermore, this genetic alteration also led to phenotypic changes, including reduced biofilm formation and siderophore production.

## Reproductive organs and polycystic ovary syndrome

Metabolites from the gut microbiota have been reported to exert regulatory effects on polycystic ovary syndrome (PCOS) (5). To investigate whether the upregulation of IL-22 in the gut microbiota mediates mitochondrial damage in granulosa cells of PCOS, Luo et al. conducted an assessment of the impact of the probiotic *Escherichia coli Nissle 1917* (EcN) on the gut microbiota-metabolism-IL-22-mitochondrial damage axis in PCOS. To achieve this, they established a PCOS mouse model using dehydroepiandrosterone and subsequently treated them with EcN, fecal microbiota transplantation (FMT), or IL-22 inhibitors. Their findings revealed that EcN alleviates mitochondrial injury in granulosa cells of PCOS by enhancing IL-22 levels, facilitating the recovery of sex hormone levels, improving ovarian tissue morphology, and promoting amino sugar and nucleotide sugar metabolism.

## Host cell endogenous drug candidates

Itaconate (ITA) is a signaling metabolite generated by classically activated macrophages (6), and its esterified derivatives, 4-octyl ITA (4-OI) and dimethyl ITA (DMI), hold promise as drug candidates against various infections. To understand the effector mechanisms of ITA, Yuk et al.’s review highlights four factors contributing to its effects: nuclear factor erythroid 2-related factor 2 (*NFR2*), activating transcription factor 3 (*ATF3*), transcription factor EB (*TFEB*), and Akt. They point out that both 4-OI and DMI are employed to overcome low cell permeability. Furthermore, 4-OI inhibits inflammation by alkylating glyceraldehyde-3-phosphate dehydrogenase (*GAPDH*) and exerts its antiviral properties through NRF2 signaling. In addition, DMI enhances protection against bacterial infections by activating NRF2 and ATF3, reducing Akt phosphorylation. However, it is likely that other contributing factors are involved, necessitating further investigation.

## Conclusion

In summary, this Research Topic demonstrates a significant interdependence between metabolic regulation and immune processes in combatting microbial infections.

## Author contributions

MJ: Conceptualization, Funding acquisition, Writing – review & editing. JY: Formal Analysis, Resources, Writing – review & editing. CZ: Formal Analysis, Visualization, Writing – review & editing. HM: Formal Analysis, Investigation, Writing – original draft.

## References

[1] Onarman Umu ÖC Fauske AK Åkesson CP Pérez de Nanclares M Sørby R Press CM. Gut microbiota profiling in Norwegian weaner pigs reveals potentially beneficial effects of a high-fiber rapeseed diet. PloS One (2018) 13(12):e0209439. doi: 10.1371/journal.pone.0209439 30571797 PMC6301702

[2] Cantu-Jungles T Hamaker B. New view on dietary fiber selection for predictable shifts in gut microbiota. MBio (2020) 11(1):e02179–19. doi: 10.1128/mbio.02179-02119 PMC7029134 32071263

[3] Munang’andu HM Evensen Ø. Correlates of protective immunity for fish vaccines. Fish Shellfish Immunol (2019) 85:132–40. doi: 10.1016/j.fsi.2018.03.060 29621636

[4] Plotkin SA. Correlates of protection induced by vaccination. Clin Vaccine Immunol (2010) 17(7):1055–65. doi: 10.1128/CVI.00131-10 PMC2897268 20463105

[5] Yurtdaş G Akdevelioğlu Y. A new approach to polycystic ovary syndrome: the gut microbiota. J Am Coll Nutr (2020) 39(4):371–82. doi: 10.1080/07315724.2019.1657515 31513473

[6] Seim GL Britt EC John SV Yeo FJ Johnson AR Eisenstein RS. Two-stage metabolic remodelling in macrophages in response to lipopolysaccharide and interferon-γ stimulation. Nat Metab (2019) 1(7):731–42. doi: 10.1038/s42255-019-0083-2 PMC7108803 32259027

